# Supplementary material for: Complement receptor 1 gene (CR1) intragenic duplication and risk of Alzheimer’s disease
Source: Hum Genet. 2018 Apr 19;137(4):305–14. doi: 10.1007/s00439-018-1883-2 (PMC5937907; doi:10.1007/s00439-018-1883-2)
Supplement: Supplementary file 1 — Supplementary material 1 (DOCX 13 kb) [file 439_2018_1883_MOESM1_ESM.docx]

**Supplementary Table 1 PRT assays for *CR1* LCR copy number**

| Assay | Forward primer 5′3′ | Reverse primer 5′3′ | Fluorescent label | Reference (*CR1*L) product size (bp) | Test (*CR1*) product size (bp) | Annealing temperature (°C) | Cycle number |
| --- | --- | --- | --- | --- | --- | --- | --- |
| PRT1 | GAGGAGACCCATAGTTCTTTACCA | CATCACCTATCACACTGGTGC | HEX | 108 | 115 | 63 | 24 |
| PRT2 | GCTGTTCCAGGGTCAGAGTTA | TTGGTCACATGATAGTCCTGC | NED | 164 | 187 | 63 | 25 |
| PRT3 | CTGTTTGAATAACTAGGTGGGAAGA | TTCCCTCCAGATCTATCTAGATCTAGA | FAM | 142 | 149 | 61 | 27 |

**Supplementary table 2 Control samples for *CR1* paralogue ratio test**

| **Sample ID** | **Population** | **Cohort** | ***CR1* LCR copy number** |
| --- | --- | --- | --- |
| NA18507 | Yoruba from Ibadan, Nigeria | HapMap, 1000 Genomes | 3 |
| NA18555 | Chinese Han from Beijing | HapMap, 1000 Genomes | 4 |
| NA18517 | Yoruba from Ibadan, Nigeria | HapMap, 1000 Genomes | 4 |
| NA19239 | Yoruba from Ibadan, Nigeria | HapMap, 1000 Genomes | 5 |
| NA18572 | Chinese Han from Beijing | HapMap, 1000 Genomes | 3 |
| C0140 | UK | HRC-1 | 5 |
| C0182 | UK | HRC-1 | 5 |
